# Efficient Degradation of Congo Red in Water by UV-Vis Driven CoMoO_4_/PDS Photo-Fenton System

**DOI:** 10.3390/molecules27248642

**Published:** 2022-12-07

**Authors:** Huimin Zhou, Yang Qiu, Chuanxi Yang, Jinqiu Zang, Zihan Song, Tingzheng Yang, Jinzhi Li, Yuqi Fan, Feng Dang, Weiliang Wang

**Affiliations:** 1 Institute of Environment and Ecology, Shandong Normal University, Jinan 250358, China; 2 School of Environmental and Municipal Engineering, Qingdao University of Technology, Qingdao 266525, China; 3 Middle School of Gantian, Chenzhou 424400, China; 4 Key Laboratory for Liquid-Solid Structural Evolution and Processing of Materials, Shandong University, Jinan 250061, China

**Keywords:** CoMoO_4_, PDS, Congo red, photo-Fenton

## Abstract

In order to improve the catalytic activity of cobalt molybdate (CoMoO_4_), a PDS-activated and UV-vis assisted system was constructed. CoMoO_4_ was prepared by coprecipitation and calcination, and characterized by XRD, FTIR, Raman, SEM, TEM, XPS, TGA Zeta potential, BET, and UV-Vis DRS. The results showed that the morphology of the CoMoO_4_ nanolumps consisted of stacked nanosheets. XRD indicated the monoclinic structures with C2/m (C^3^_2h_, #12) space group, which belong to α-CoMoO_4_, and both Co^2+^ and Mo^6+^ ions occupy distorted octahedral sites. The pH of the isoelectric point (pHIEP) of CMO-8 at pH = 4.88 and the band gap of CoMoO_4_ was 1.92 eV. The catalytic activity of CoMoO_4_ was evaluated by photo-Fenton degradation of Congo red (CR). The catalytic performance was affected by calcination temperature, catalyst dosage, PDS dosage, and pH. Under the best conditions (0.8 g/L CMO-8, PDS 1 mL), the degradation efficiency of CR was 96.972%. The excellent catalytic activity of CoMoO_4_ was attributed to the synergistic effect of photo catalysis and CoMoO_4_-activated PDS degradation. The capture experiments and the ESR showed that superoxide radical (·O_2_^−^), singlet oxygen (^1^O_2_), hole (h^+^), sulfate (SO_4_^−^·), and hydroxyl (·OH^−^) were the main free radicals leading to the degradation of CR. The results can provide valuable information and support for the design and application of high-efficiency transition metal oxide catalysts.

## 1. Introduction

**Figure 1:**
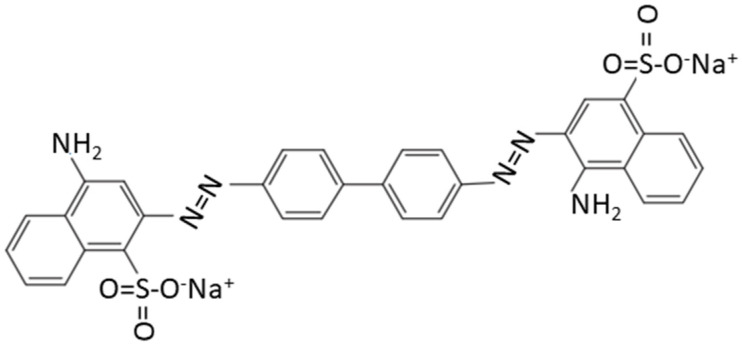
Molecular structure of CR.

Congo red (CR) is an azo dye in organic dyes (Figure 1), which is widely used in papermaking, plastics, cosmetics, pharmaceuticals, and other fields. However, its widespread use also brings a variety of problems, such as reduced visibility of water, resulting in water ecological environment problems [1,2,3]. It is also harmful to human health, owing to its teratogenicity and carcinogenic potential [4,5,6,7]. Thus, it is of great significance to find an effective way to treat CR-polluted wastewater.

In general, membrane filtration processes, precipitation, coagulation, biological treatment, adsorption, ion exchange, electrochemical processes, advanced oxidation processes (AOPs), and ozonation are suitable choices [8,9,10,11,12,13,14]. AOPs were considered to be an effective method for degrading organic dye wastewater [15]. Fenton technology has been widely applied to the degradation of organic dye wastewater. However, the low pH, low utilization of oxidant, large amount of reagent, and the formation of an iron sludge precipitation limit its wide application [16]. In addition to Fenton oxidation, persulphate oxidation as a type of AOPs has also attracted much attention in the field of wastewater treatment. Persulfate oxidation can produce strong oxidizing sulfate radicals (SO_4_·^−^) under the different activation factors to degrade many organic compounds, presenting the advantages of strong oxidation ability (E^0^ = 2.6–3.1 V), wide pH application range (2.0–10.0), and long half-life [17]. Recent studies have shown that cobalt ions and their composites can more effectively decompose the peroxydisulfate (PDS, S_2_O_8_^2−^) to form the free radical species [18]. Furthermore, the electron transition in Co^2+^ and Co^3+^ can also assist in the generation of radicals and non-radicals. As a highly effective photo-Fenton catalyst to decompose RhB under visible light, Li-prepared Zn/Co-ZIFs@MIL-101 (Fe) composites demonstrated a remarkable capability to remove RhB [19]. Among transition metal oxides, molybdenum oxide is a promising candidate for such applications because of its crystal structure, multiple oxidation states, N-type semiconductors, and reversible small ion storage. Molybdenum atoms in molybdate have a variety of coordination modes and redox activities, and molybdate is widely used in optical, electrochemical, magnetic, antimicrobial, and other functional materials [20,21,22,23,24]. Molybdate can be prepared by various simple methods, including hydrothermal synthesis, coprecipitation, and so on [25,26,27,28]. It is very noteworthy that these characteristics also cater to PDS activation. 

CoMoO_4_ as a promising electrochemical material due to its stable crystal structure, high conductivity, fast electron transmission, and high redox ability, also shows good catalytic activity in PMS activation for organic degradation [29]. In addition, CoMoO_4_ can complete photogenerated electron transition under UV-vis irradiation, but the hole-electron recombination is easy [30]. Previous studies of CoMoO_4_ as a photo-catalyst are shown in Table 1 [29,31,32,33,34]. In comparison with PMS, PDS has drawn growing attention due to its high solubility and stability under mild conditions. Accordingly, we chose PDS to construct the CMO-PDS system [35]. Therefore, wolframite cobalt molybdate catalyst was successfully synthesized by the coprecipitation and calcination method. After activation by persulfate (PDS), the CR dye solution was degraded by photocatalytic reaction.

In this study, CoMoO_4_ was prepared by co-precipitation and calcination, and characterized by XRD, FTIR, Raman, SEM, TEM, XPS, TGA, DTG, Zeta, BET, UV-Vis DRS, and ESR. Due to the excellent performance of CoMoO_4_ for organic dye pollutant degradation, we chose it as a catalyst for the degradation of CR dye wastewater. The photo-Fenton degradation of CR, capture experiment to evaluate the activity of the catalyst, and the photo-Fenton catalytic activity of the catalyst were affected by calcination temperature, catalyst dosage, PDS dosage, and pH. The capture experiment and ESR verified that ·O_2_^−^, ·OH, SO_4_^−^·, h^+^, and ^1^O_2_ were the main active species (ROS). The experimental results indicated that the transition metal oxides provided support for the degradation of organic dyes. The results can provide valuable information and support the design and application of high-efficiency transition metal oxide catalysts.

## 2. Experiment

### 2.1. Chemicals and Materials

Ammonium persulfate (PDS; 98.5%), Congo red (CR; 99%), sodium molybdate dihydrate (Na_2_(MoO_3_)_3_·2H_2_O; 99.95%), cobalt chloride hexahydrate (CoCl_2_·6H_2_O; 99.95%), p-benzoquinone (p-BQ; 99%), L-histidine (99.5%), and ammonium oxalate monohydrate (AO; 99.99%) were purchased from Shanghai Macklin Biochemical Co., Ltd. (Shanghai, China). Sodium molybdate dihydrate (Na_2_MoO_4_·2H_2_O; 99%), ammonia solution (NH_4_OH), sulfuric acid (H_2_SO_4_), methanol (MeOH), ethanol (EtOH) and tert-butyl alcohol (TBA) were purchased from Sinopharm Chemical Reagent Co., Ltd. (Shanghai, China). All reagents and chemicals used in this study were of analytical grade and were used without further purification.

### 2.2. Preparation of Samples

CoMoO_4_ samples were synthesized by co-precipitation and calcination, and the specific process was as follows: CoCl_2_·6H_2_O and NaMoO_4_·2H_2_O were used as Co and Mo sources, respectively. We dissolved 1 g of CoCl_2_ **·** 6H_2_O in 20 mL distilled water and stirred at 70 °C; then, 30 mL deionized water containing 1.02 g of dissolved Na_2_MoO_4_ **·** 2H_2_O was added and the solution was stirred for 5 h. The purple solid obtained was centrifuged and dried at 120 °C overnight. The initial product was ground into powder, heat-treated under air atmosphere for 180 min at 800 °C (900, 1000 °C) at a heating rate of 2 °C min^−1^ and naturally cooled to room temperature. The obtained products were named as CMO-8, CMO-9, and CMO-1.

### 2.3. Characterization

Powder X-ray diffraction (XRD) patterns of the as-prepared samples were obtained at room temperature with a D/MAXRC X-ray diffractometer using Cu Ka radiation source which operated at 45 kV and 40 mA. The structure and morphology of the sample was performed by scanning electron microscopy (SEM, Zeiss Gemini 300, Gena, Germany) and transmission electron microscopy (TEM, JEOL JEM-2100F Japan). Raman spectra were obtained using a confocal Raman microscope Horiba LabRAM HR (Bruker, Billerica, MA, USA) excited by a laser source of 10 W at a specific λ of 325 nm. The Fourier transform infrared (FTIR) spectra were collected on a ThermoScientific Nicolet IS5 Fourier Transform infrared spectrometer (Waltham, NJ, USA) in the wavenumber range of 400–4000 cm^−1^ resolution through the KBr pellet method. Thermal analysis was studied by a Netzsch TG209F3 (Netzsch TG209F3, Bayern, Germany) at a heating temperature ramping rate of 10 °C min^−1^ in the temperature range of 40–800 °C under air/N_2_ atmosphere. The pH of the isoelectric point (pHIEP) of materials was determined by using a Zetasizer Nano analyzer (Malvern zetasizer nano ZS, Malvern, UK). The Brunauer-Emmett-Teller (BET) specific surface area of the sample was measured using the Autosorb-iQ instrument (Tristar II 3020, USA). The UV-vis diffuse reflectance spectra (UV-vis DRS) of the samples were measured by UV-vis spectrophotometer (UV3600PLUS, Japan). The electron spin resonance (ESR) measurements were performed on a Bruker EMXnano spectrometer (EMX10/12, Bruker, Germany). The intermediate products of CR degradation were identified by high-performance liquid chromatography equipped with mass spectrometry (Thermo Scientific™ Q Exactive™, Waltham, MA, USA).

### 2.4. Catalytic Activation Experiments

We dispersed 0.8 g/L of catalysts in a 50 mL solution containing 100 mg/L of CR. The solution was kept in the dark for 60 min for adsorption saturation to be achieved, and the catalyst was dispersed in the water in the form of nanoplatelets. A 500 W xenon lamp was selected. Degradation was initiated during the UV-vis process by adding PMS solution (0.5 mM) to the suspension. The solution was stirred by a magnetic stirrer at room temperature for the reaction. To analyze catalytic activity, 1 mL of the suspension was sampled within a given time interval. The number of sampling times and time intervals depended on the degradation rate. Each sample was quenched with 1 mL methanol and filtered through a 0.22 µm membrane filter for further analysis. To test the reusability of the material, after measurements, the catalysts were recycled by centrifugation, washed with deionized water and ethanol, and then dried in an oven at 60 °C to further investigate their reusability. 

## 3. Results and Discussion

### 3.1. Characterization of CoMoO_4_

**Figure 2:**
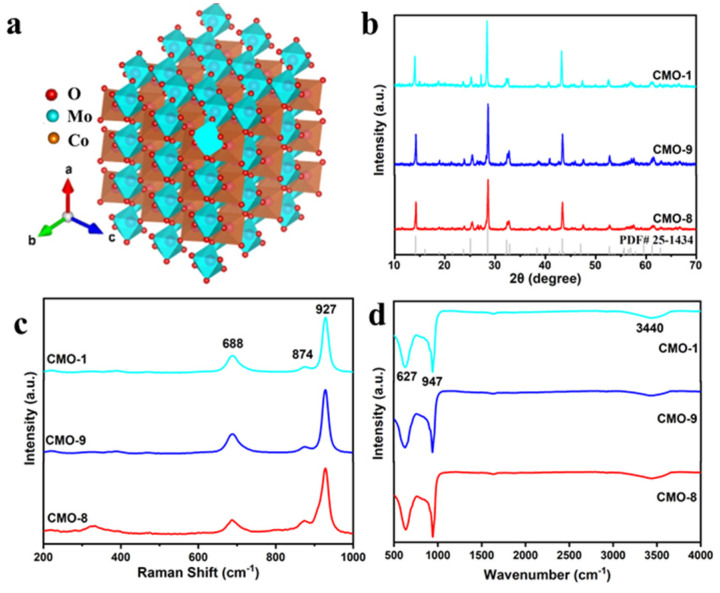
(**a**) Determined unit crystal illustration (cyan represents Mo, jacinth represents Co, and red represents O); (**b**) XRD; (**c**) Raman; (**d**) FTIR spectra of CMO-8, CMO-9, and CMO-1.

We selected CoMoO_4_ with ABO_4_ wolframite as the photocatalyst; Co ions occupy the A six-coordinated, whereas Mo ions occupy the B six-coordinated as show in Figure 2a. The crystal phase of the resultant samples was identified by X-ray diffraction (XRD). As shown in Figure 2b, the diffraction peaks of all samples are consistent with the monoclinic phase of CoMoO_4_ (JCPDS 25-1434) [36]. With the increase of temperature, the growth of crystal will be changed to some extent. Thus, the XRD spectra of CMO-1 show an intense peak around 27° compared with CMO-8 and CMO-9 [36]. In this form, both Co^2+^ and Mo^6+^ ions occupy distorted octahedral sites [37]. The calculated lattice parameters, a = 9.628 Å, b = 8.865 Å, c = 7.694 Å, b = 112.62, and cell volume = 606.19 Å^3^, for the heated α-CoMoO4 sample match well with JCPDS 025-1434, confirming the formation of a pure monoclinic α-CoMoO4, C2/m space group [38]. In α-CoMoO4, the main characteristic peaks are obtained at 2θ of 14.159° (110), 25.063° (002), 32.267° (−222), 28.512° (220), 32.267° (−222), and 43.340° (−330), and these can be indexed with JCPDS 025-1434. There are two types (α phase and β phase) of molybdate, such as CoMoO_4_, FeMoO_4_, and NiMoO_4_ [36,37]. 

Raman scattering was observed at 688, 874, and 927 cm^−1^ for CoMoO_4_, as shown in Figure 2c. A strong band at 927 cm^−1^ and two weak bands centered around 874 cm^−1^ were the characteristic bands of CoMoO_4_ representing the Mo–O–Co vibrational stretching frequencies [39]. The FTIR spectra of CoMoO_4_ are shown in Figure 2d. For pure CoMoO_4_, the bands in the range of 750–950 cm^−1^ are attributed to the stretching vibration of Mo–O bonds of distorted MoO_4_ in CoMoO_4_ [40,41]. The bands in the lower frequency range (400–700 cm^−1^) belong to Co–Mo–O stretching vibrations. The spectra at 613 and 950 cm^−1^ correspond to the Co−O and Mo−O−Mo vibration modes. It is believed that the absorption peak at 3441 cm^−1^ can be attributed to the O–H stretching vibration and its corresponding O–H bending vibration occurs at 1636 cm^−1^, due to chemically adsorbed water molecules; the vibration band at around 947 cm^−1^ is attributed to the activation of υ1 vibration of the distorted MoO_4_ tetrahedron present in CoMoO_4_ [42].

**Figure 3:**
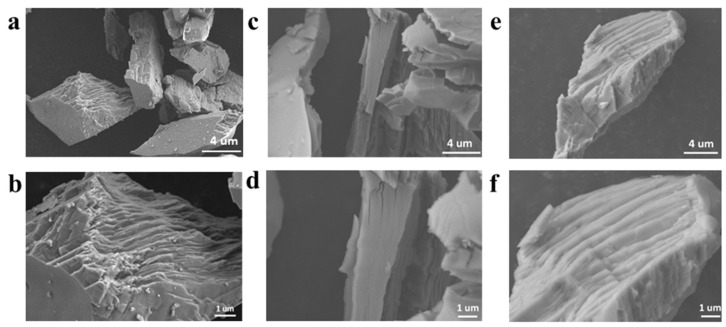
SEM images of CMO-8 (**a**,**b**); CMO-9 (**c**,**d**); CMO-1 (**e**,**f**).

The SEM images of CMO-8, CMO-9, and CMO-1 are shown in Figure 3a–f, respectively. The nanolumps consist of stacked nanosheets, which expose a large number of active sites and provide sufficient space for CR to attach. The rising temperatures cause nanosheets to agglomerate in a way that makes them more compact with each other; accordingly, at 1000 °C, the nanosheets stack more tightly [43]. Thus, the degradation of CR for CMO-1 was poor compared with CMO-8 and CMO-9.

**Figure 4:**
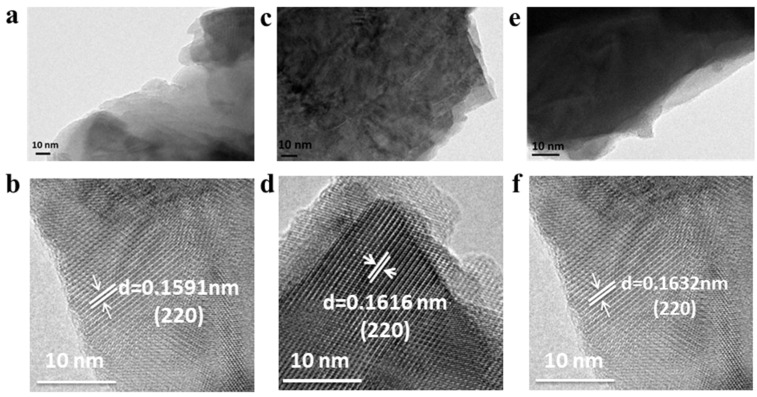
TEM images of CMO-8 (**a**,**b**); CMO-9 (**c**,**d**); CMO-1 (**e**,**f**).

The images of SEM can also be indicated by TEM. Figure 4a–f represent the HRTEM images of CMO-8, CMO-9, and CMO-1, respectively. The TEM images of CMO-8, CMO-9, and CMO-1 provide a clear view of the morphological shape of the nanolumps that consist of nanosheets as the temperature increase. The spacings of the lattice fringes of CMO-8, CMO-9, and CMO-1 are measured to be 0.1591, 0.1616, and 0.1632 nm, respectively, which can be ascribed to the (220) plane of α-CoMoO_4_, and both match well with the monoclinic phase of CoMoO_4_ (JCPDS 25-1434). The calcination temperature did not change the crystal structure, and the lattice spacing measured by the digital micrograph was consistent with the XRD crystal structure.

**Figure 5:**
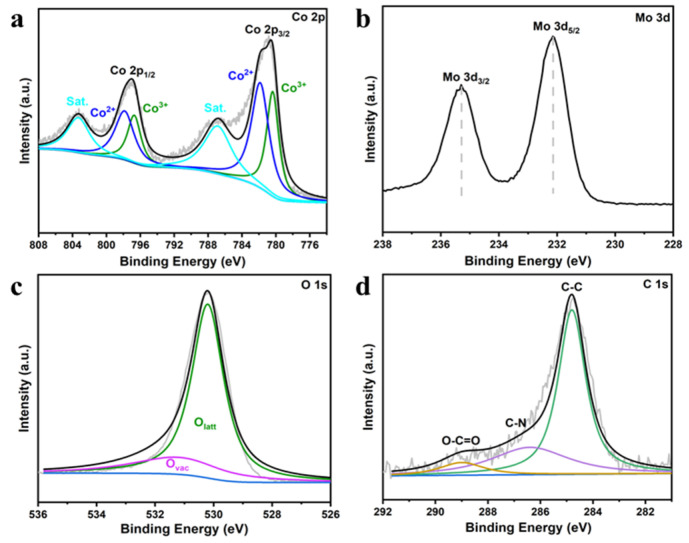
XPS spectra of (**a**) Co 2p; (**b**) Mo 3d; (**c**) O 1s; (**d**) C 1s of CMO-8.

The surface composition and chemical states of the CoMoO_4_ were further characterized by XPS. The full-survey-scan spectra further demonstrate the presence of Co, Mo, O, and C (Figure 5 and Figure S1). Figure 5a shows the curves of Co 2p, which shows two obvious peaks of 2p3/2 and 2p1/2, and two satellites (marked as “Sat.”). Two low binding energy peaks at 780.35 and 796.7 eV are assigned to Co^3+^, while the other peaks at 781.82 and 797.0 eV are well-matched with Co^2+^ [44,45]. Figure 5b displays Mo 3d XPS doublets, which correspond to Mo 3d_5/2_ and Mo 3d_3/2_. The Mo 3d doublet binding energies of CoMoO_4_ shift towards a lower level. The peaks at 232.2 and 235.25 eV come from the Mo^6+^ [45]. As shown in Figure 5c, the peak at 530.18 eV was ascribed to the lattice oxygen (O_latt_) bonds of metal oxides; the peak at 531.28 eV might be owing to the defect site of low hypoxia coordination, indicating that O_vac_ were created on the surface of CoMoO_4_ [46]. The ratios of O_latt_ and O_vac_ were 77.773% and 22.227%. Figure 5d shows the C1s: the peak at 284.8 eV was ascribed to the C-C, the peak at 286.4 eV was ascribed to the C-N, and the peak at 289 eV was ascribed to the O-C=O [47]. Figure S1 shows the survey spectra. This further confirms the formation of CoMoO_4_.

**Figure 6:**
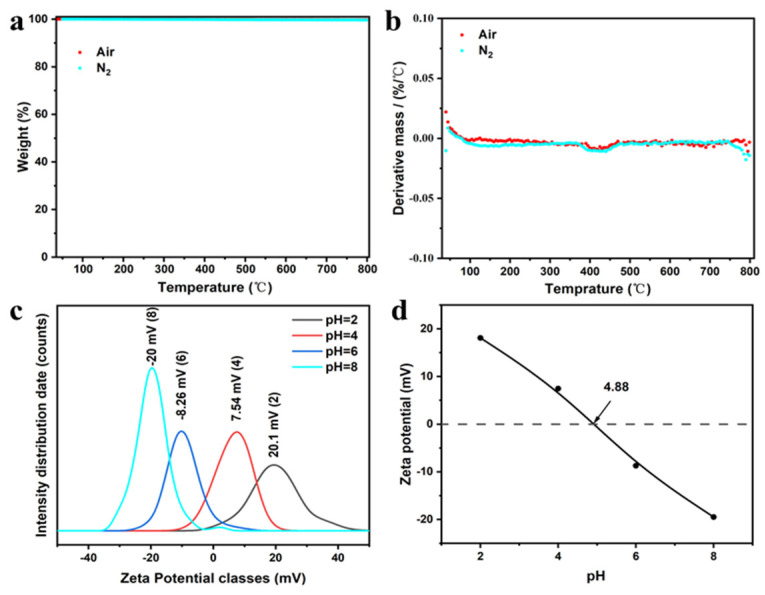
(**a**) TGA; (**b**) DTG; (**c**) Zeta potential of various pH with 2, 4, 6, and 8; (**d**) The variation curve for zeta potential as a function of pH value of CMO-8.

CMO-8 samples were heated from 40 °C to 800 °C under an air/N_2_ flow of 20 mL/min, and the heating rates were 10 °C/min. In order to eliminate systematic error, blank tests were performed before the experiment to provide the baseline, and repetitions were also performed to ensure good reproducibility of the results. Figure 6a,b shows the TGA and DTG curves of CMO-8 at 10 °C/min, with the 0.23682% and the 0.36296% of weightlessness in air and N_2_ atmosphere, respectively. The CMO-8 sample exhibits excellent thermal stability in both air and N_2_ atmospheres due to its negligible mass decrease.

Figure 6c shows the variation in the zeta potential of the CMO-8 sample at different pH. The Zeta potential classes are −20 mV, −8.26 mV, 7.54 mV, and 20.1 mV at pH = 8, pH = 6, pH = 4, and pH = 2, respectively. Figure 6d shows the pH of the isoelectric point (pHIEP) of CMO-8 at pH = 4.88. When pH < 4.88, the adsorbent is positively charged; when pH > 4.88, the adsorbent is negatively charged.

**Figure 7:**
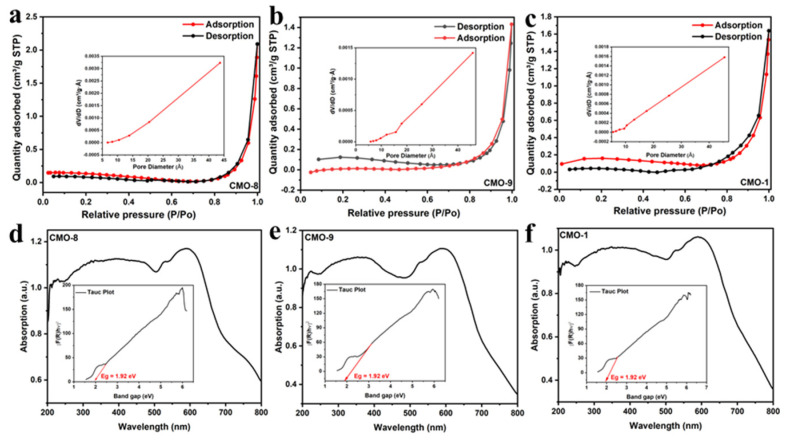
BET of (**a**) CMO-8; (**b**) CMO-9; (**c**) CMO-1; UV-visible diffuse reflectance spectra of (**d**) CMO-8; (**e**) CMO-9; (**f**) CMO-1.

Figure 7a–c showed the BET of CMO-8, CMO-9, and CMO-1, respectively. The specific surface area of CMO-8, CMO-9, and CMO-1 were 0.5451, 0.5186, and 0.4531 m^2^/g, respectively. Among them, the CMO-8 exhibited the highest surface area and pore volume, which may be beneficial to the diffusion and adsorption of CR molecules and expose more active sites to activate PDS to generate SO4^−^· and ·OH.

To determine the band gap energy of semiconductor materials, two prominent methods are used: (i) Tauc method: α = A (hv-Eg) ^2^/λ for direct (allowed) and α = A (hv-Eg) ^1/2^/λ for indirect (allowed) (α = absorption coefficient; A = absorption constant for indirect transitions depending on the transition probability) [48,49,50,51,52]. Figure 7d–f shows the UV-visible diffuse reflectance spectra (DRS) of CoMoO_4_. The optical band gap energy (Eg) of CMO-8, CMO-9, and CMO-1 were both found to be 1.92 eV, which is in accordance with the value already published [53].

### 3.2. Degradation of CR in Different Condition

The initial concentration of CR was 100 mg/L in all experiments. All experiments were carried out under UV-vis after 1h adsorption in dark conditions, and PDS (0.5 mmol/L) was added after adsorption. The number of sampling times and time intervals depended on the degradation rate.

**Figure 8:**
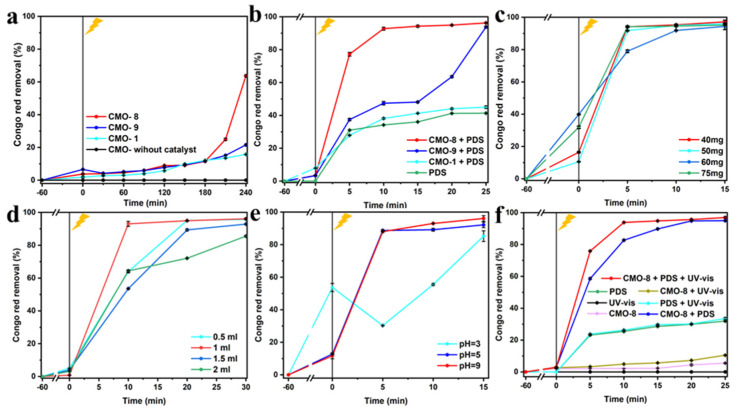
Degradation of CR of (**a**) CMO-8, CMO-9, CMO-1; (**b**) CMO-8, CMO-9, CMO-1 + PDS; (**c**) Effect of catalyst loading; (**d**) Effect of PDS concentration; (**e**) Effect of pH; (**f**) Degradation of CR in different conditions. The error bars represent the standard deviation (n = 3).

Figure 8a,b shows that the dosage of CMO-8, CMO-9, and CMO-10 was 20 mg. Figure 8a shows that without PDS, the removal rates of CR were 63.68% (63.68% ± 0.555%), 21.561% (21.561% ± 0.12%), and 15.753% (15.753% ± 0.145%) under UV-vis after 240 min, respectively. Figure 8b shows that the removal rates of CR were 96.29% (96.29% ± 0.158%), 93.782% (93.782% ± 0.446%), and 45.024% (21.561% ± 0.12%) after the addition of 1 mL PDS under UV-vis for 25 min, respectively. This indicated that there is an excellent synergy between the CoMoO_4_ catalyst and PDS, and this is more noticeable with CMO-8. The removal of CR with CMO-9 and CMO-1 were poor compared with CMO-8, which were closely consistent with their morphologies. Thus, we chose CMO-8 as the optimal photocatalyst for subsequent experiments.

Figure 8c shows the degradation of CR by CMO-8 at different concentrations ranging from 0.8–1.5 g/L. At dosages of 40 mg (0.8 g/L), 50 mg (1 g/L), 60 mg (1.2 g/L), and 75 mg (1.5 g/L) with 1 mL PDS, the removal rates of CR were 97.169% (97.169% ± 1.051%), 95.982% (95.982% ± 0.185%), 94.38% (94.38% ± 2%), and 95.147% (95.14% ± 0.129%) after 15 min, respectively. Therefore, we chose the optimal dosage of 40 mg for subsequent experiments. Figure S2a shows the removal of CR by reused CMO-8 at different dosages of concentrations ranging from 40–75 mg (0.8–1.5 g/L). At dosages of 40 mg (0.8 g/L), 50 mg (1 g/L), 60 mg (1.2 g/L), and 75 mg (1.5 g/L) with 1 mL PDS, the removal rates of CR were 73.181%, 79.203%, 82.798%, and 81.05% after 40 min of UV-vis, respectively. Therefore, we chose the optimal dosage of 60 mg for subsequent experiments. This result indicated that the more PDS was required to active reused CMO-8.

Figure 8d shows the degradation of CR with a CMO-8 dosage of 40 mg; different dosages of PDS ranging from 0.5–2 mL were studied. As the dosage was increased from 0.5 mL, 1 mL, 1.5 mL, and 2 mL with 0.8 g/L catalyst, the removal rates of CR were 95.776% (95.776% ± 0.139%), 96.121% (96.121% ± 0.594%), 92.874% (92.874% ± 0.1%), and 85.526% (85.526% ± 0.402%) after 30 min of UV-vis, respectively. When the dosage of PDS was below 1 mL, the removal of CR was increased. In contrast, when the dosage of PDS was over 1 mL, the removal of CR was decreased. This is closely related to the degree of activation of the dosed catalyst. Therefore, we chose a dosage of 1 mL of PDS as the best option for subsequent studies. Figure S2b shows the removal of CR with a dosage of 60 mg at different concentrations of PDS ranging from 0.5–2 mL. At concentrations of 0.5 mL, 1 mL, 1.5 mL, and 2 mL with 60 mg (1.2 g/L) of reused catalyst, the removal rates of CR were 48.03%, 64.184%, 67.275%, and 68.367% after 30min of UV-vis, respectively. Therefore, we chose the dosage of 2 mL of PDS as the best option in subsequent studies of the reused catalyst. As the dosage of PDS increased, the removal rate of CR increased. Due to the secondary utilization of CMO-8, the synergistic effect was weakened. Thus, the CMO-8 activation required more dosage of PDS.

CR is an anionic dye in nature and at neutral pH, it appears as a red color in aqueous solutions. The color of CR changes with the solution pH. At low pH, it appears blue due to tautomerism. Thus, the adsorption of CR onto CMO-8/PDS was studied at pH 3–9. Figure 8e shows the degradation of CR by 40 mg CMO-8 and 1 mL PDS at different pH. When pH = 3, 5, and 9, the degradation efficiencies of CR were 85.203% (85.203% ± 3.281%), 92.16% (92.16% ± 1.677%), and 96.077% (96.077% ± 1.764%) after 15 min of UV-vis, respectively. With the increasing pH, CMO-8 degradation toward CR increased. Under alkaline conditions, CR is an anionic dye with a positive charge due to a higher solution concentration of OH^−^. Due to mutual attraction between positive and negative charges, CR can be more quickly adsorbed and decomposed at pH = 9, relative to pH = 3 and 5. The degradation efficiency was excellent. Under subsequent illumination, the concentration increased due to desorption and side reactions. When the pH is decreased, the adsorption was poor. We evaluated the Zeta potential to estimate the surface charge of CMO-8 to better understand the effect of pH on the adsorption process. Figure S2c shows the degradation of CR with 60 mg of the reused CMO-8 catalyst and 2 mL of PDS at different pH. When pH = 3, 5, and 9, the degradation efficiencies of CR were 88.504%, 92.001%, and 96.955%, respectively, after 35 min of UV-vis. These results further validate the above conclusions.

Figure 8f shows the degradation of CR in UV-vis, CMO-8, UV-vis with CMO-8, PDS, PDS with UV-vis, CMO-8 with PDS, and CMO-8 with PDS and UV-vis; the corresponding degradation was 0, 5.519% (5.519% ± 0.207%), 10.486% (10.486% ± 0.168%), 31.922% (31.922% ± 0.218%), 33.532% (33.53% ± 0.551%), 94.989% (94.989% ± 0.1%), and 96.972% (96.972% ± 0.5%) after 25 min, respectively. In this study, it can be concluded that there is a good synergy between the CMO-8 catalyst and PDS, and with the participation of UV-vis, the degradation effect of the whole system on CR is greatly improved. Thus, the CMO-8/PDS-UV-vis system for CR degradation was successful.

### 3.3. Degradation Mechanism by CMO under PDS Activation and Visible Light Irradiation

**Figure 9:**
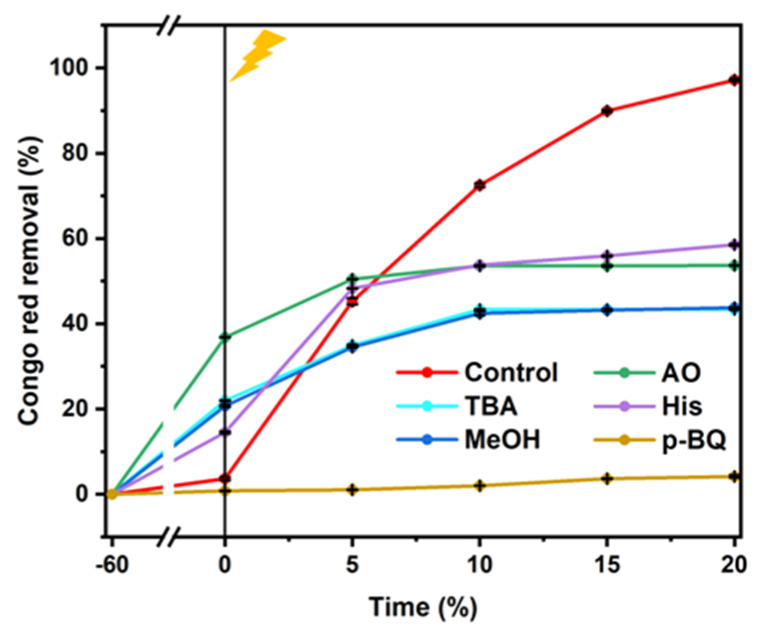
Effect of radical scavengers on the removal of CR in the CMO-8/PDS system (conditions: 100 mg·L^−1^ CR, 1 g·L^−1^ catalyst, 0.5 mM PMS and pH of 7). The error bars represent the standard deviation (n = 3).

Generally speaking, ROS, such as ·OH, SO_4_·^−^, O_2_·^−^, and ^1^O_2_, play important roles in the oxidation of organic pollutants in PDS-based AOPs. To confirm the presence of ROS in the CoMoO_4_/PDS system, various quenching tests were deduced. As is reported, MeOH could be used as a capture compound for the total flux of ·OH and SO_4_·^−^ because of its high reactivity with both of these species (k OH/MeOH = 9.7 × 10^8^ M^−1^s^−1^; kSO_4_·^−^/MeOH = 1.6 × 10^7^ M^−1^s^−1^) [54,55]. The p-BQ was also chosen as scavengers for HO_2_·/O_2_·^−^ with a rate constant of (k O_2_·^−^/p-BQ = 1.0 × 10^9^ M^−1^s^−1^) [56]. TBA was used as a special ·OH capture because TBA reacts with ·OH approximately 1000 times greater than it reacts with SO_4_·^−^ (k·OH/TBA = (3.8–7.6) × 10^8^ M^−1^s^−1^; kSO_4_·^−^/TBA = (4–9.1) × 10^5^ M^−1^s^−1^) [57]. In addition, L-histidine (L-His, ^1^O_2_ scavenger, K^1^O_2_ = 1.2 × 10^8^ M^−1^ S^−1^) was used [55]. Figure 9 shows that the degradation of CR was significantly suppressed from 97.168% (97.168% ± 0.3%) (control) to 43.33% (43.33% ± 0.181%), 43.739% (43.739% ± 0.135%), 53.684% (53.684% ± 0.124%), 58.507% (58.507% ± 0.21%), and 4.17% (4.17% ± 0.331%) with the addition of 1 mM TBA, 1 mM MeOH, 1 mM (NH_4_)_2_C_2_O_2_, 1 mM His, and 1 mM p-BQ to the system, respectively. Therefore, ·OH, SO4^−^·, h^+^, ^1^O_2_, and ·O_2_^−^ were the active substances in the CMO-PDS-UV-vis system. ·O_2_^−^ showed the strongest effect among five active species (·O_2_^−^> ·OH > SO_4_^−^· > h^+^ > ^1^O_2_).

**Figure 10:**
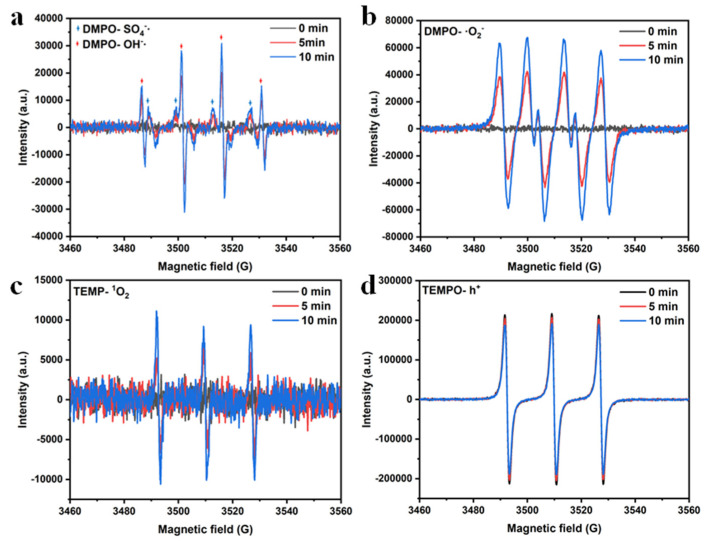
ESR analysis for the CMO-8/PDS system in aqueous dispersion by spin trapping with DMPO (**a**) SO4^−^·, OH·; (**b**) ·O_2_^−^; (**c**) TEMP-^1^O_2_; (**d**) TEMPO-h^+^ at different time intervals.

To further verify the free radical and non-radical species mentioned above, Figure 10a–d show that electron spin resonance (ESR) technology was applied to verify the quenching test [50]. Figure 10a–c show that when DMPO was added to the CMO-8/PDS reaction system for 5 min, the signal peaks of DMPO-SO_4_^−^·, DMPO-·OH, and DMPO- O_2_^−^· can be observed, confirming the generation of SO_4_^−^·, ·OH, and O_2_^−^· radicals. The intensity of the characteristics peaks of DMPO-SO_4_^−^·, DMPO-·OH, and DMPO- O_2_^−^· became stronger when DMPO was added for 10 min. Figure 10c shows that after adding TEMP to the system, a series of ^1^O_2_-based characteristic signals were found, indicating that ^1^O_2_ was also involved in the catalytic reaction, which was the non-radical oxidation pathway of the CMO-8/PDS system. Figure 10d shows that the peak intensity of the CMO-8-vis-PDS system (Figure 10d 5 min/10 min) was weaker than that of the CMO-8-vis-PDS (Figure 10d 0 min). The peak strength of the CMO-8-vis-PDS system decreased with time. The reason for these phenomena was that the h^+^ generated by photoexcitation of CMO-8 was captured by TEMPO to generate TEMPOH without paramagnetism, which reduced the intensity of the EPR spectrum. Moreover, after adding TEMPO, these observations were consistent with the studies on PDS, clearly indicating that CMO-8 can effectively activate PDS through radical and non-radical oxidation pathways to degrade CR. These results demonstrate that ·O_2_^−^, ·OH, SO_4_^−^·, h^+^, and ^1^O_2_ were very reactive oxygen species (ROSs) to degrade the dye pollutants in the CMO-8-vis-PDS system.

**Figure 11:**
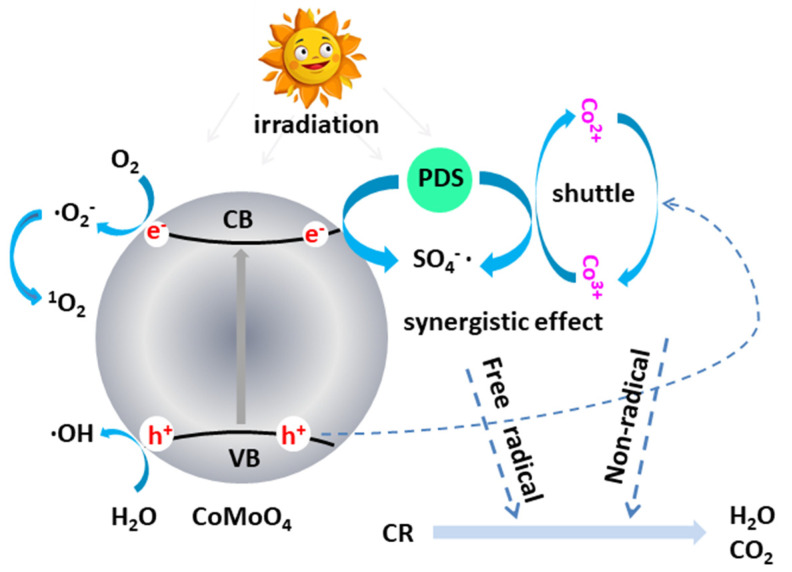
Photo-Fenton mechanism of CoMoO_4_/PDS system.

Figure 11 shows the detailed mechanism of radical species generation and transport in the CMO-vis-PDS system. During the photoactivation process, the CMO photo catalyst was activated by visible light, generating photoexcited e^−^ and h^+^ pairs when electrons were excited from the VB to the CB of CMO (Equation (1)). SO^4−^· radicals were produced from S_2_O_8_^2−^ by gaining e^−^ from the CB (Equation (2)), ·O^2−^ radicals were produced from O_2_ by gaining e^−^ (Equation (4)), and the ·OH radicals were produced by reacting with ·O^2−^, h^+^, and H_2_O (Equations (6) and (7)) [57,58]. Thus, the photo-generated h^+^, SO_4_^−^·, ·O^2−^, e^−^, and ·OH radicals participated in the degradation process. At the same time, PDS was activated by Co^2+^, producing SO_4_^−^· radicals. Combined with the abovementioned discussion in Figure 8, the excellent degradation performance of CMO could be attributed to the reactive ·O_2_^−^ species, which was mainly produced from the activation of PDS by CMO (Equation (8)) [59]. Furthermore, it can be seen from Equations (9) and (11) that the Co element could undergo a reciprocal transformation between Co^3+^ and Co^2+^ during the reaction process. Electron shuttling facilitates the circulation between Co^3+^ and Co^2+^. The possible photocatalytic and persulfate activation and equations mentioned above are listed below.

CoMoO_4_ (photo catalyst) + h v **→** e^−^ + h^+^ (1)

S_2_O_8_^2−^ + e^−^ **→** SO_4_^−^· + SO_4_^2−^ (2)

SO_4_^−^· + H_2_O **→** HSO_5_^−^ + ·OH + H^+^ (3)

O_2_ + e^−^ **→** ·O_2_^−^ (4)

·O_2_^−^ + ·OH **→**
^1^O_2_ + OH^−^ (5)

H_2_O + ·O_2_^−^
**→** ·OH (6)

H_2_O/OH^−^ + h^+^
**→** ·OH + H^+^ (7)

Co^2+^ + HSO_5_^−^
**→** Co^3+^ + SO_4_^−^· + OH^−^ (8)

Co^3+^ + HSO_5_^−^
**→** Co^2+^ + SO_5_^−^· + H^+^ (9)

HSO_5−_ + SO_5_^−^· **→** SO_4_^−^**·** + HSO_4−_ + ^1^O_2_ (10)

Co^3+^ + e^−^
**→** Co^2+^ (11)

CR + radicals (·O_2_^−^,·OH, SO_4_^−^·, h^+^, ^1^O_2_) **→** degraded products +CO_2_ + H_2_O (12)

**Figure 12:**
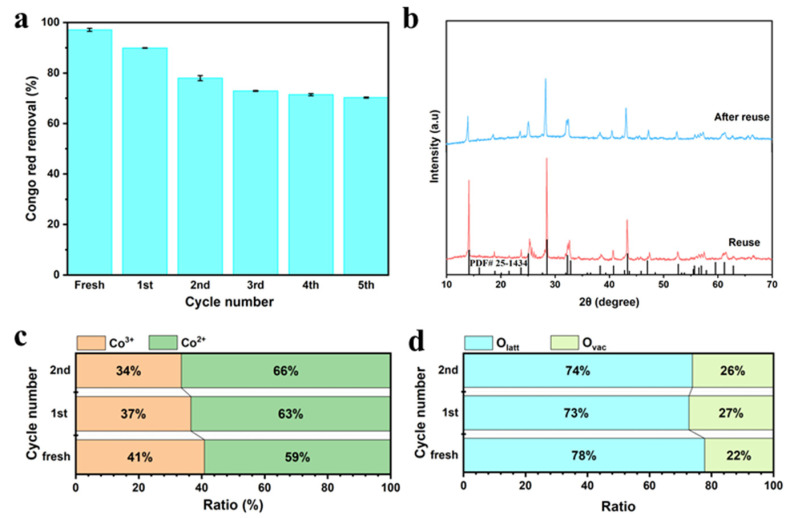
(**a**) Cyclic degradation performance, the error bars represent the standard deviation (n = 3); (**b**) the XRD of reused CMO-8 and after reuse CMO-8; the ratio of Co^2+^, Co^3+^, O_latt_, O_vac_ (**c**,**d**).

The reusability of materials is significant in measuring the economic feasibility and propensity of secondary pollutants [60]. Figure 12a shows the CR removal rate for 6 cycles to measure the reusability of CMO-8. Before reusing, we used EtOH and deionized water to wash used CMO to remove the adsorbed PDS. Compared with the first cycle (97.7% ± 0. 6%), the removal efficiency of CR in the 2nd (89.9% ± 0.16%), 3rd (77.968% ± 1%), 4th (72.901% ± 0.18%), 5th (71.433% ± 0.41%), and 6th (70.27% ± 0.244%) cycle decreased by 7.203% (±0.6%), 19.235% (±1%), 24.302% (±0.18%), 25.77% (±0.41%), and 26.933% (±0.244%), respectively. Thus, the CMO-8/PDS-UV-vis system exhibits excellent performance in CR degradation. Figure 12b shows the XRD of reused CMO-8; after reuse, the peaks at 14.159° (110), 28.512° (220), and 43.340° (−330) were the main characteristic peaks that had a sharp decline. The XRD data of the reused CMO-8 and after reuse CMO-8 are compared with the unreacted catalyst; the results showed that the main peak shape had not changed, indicating the stability of the CMO-8 composite catalyst structure before and after the reaction (the crystal shape had not changed). In summary, the stability of both the performance and structure reflected the good cyclability and stability of the reaction system. Thus, it can be indicated that the reason of the activity liveness competence of CMO-8 decreased. Therefore, this is the reason that the degradation of CR expressed a gradual decline downdrift.

Figures S3 and S4 show the XPS spectra of reused CMO-8 and after reuse CMO-8. Figure S3a shows the curves of Co 2p, which shows two obvious peaks of 2p_3/2_ and 2p_1/2_, and two satellites (marked as “Sat.”). Two low binding energy peaks at 780.38, 780.4 and 796.71, 796, 73 eV are assigned to Co^3+^, while the other peaks at 781.81, 781.8 and 797.85, 797.85 eV are well-matched with Co^2+^. Figure S3b displays Mo 3d XPS doublets, corresponding to Mo 3d_5/2_ and Mo 3d_3/2_. The peaks at 232.35, 232.45 and 235.5, 235.6 eV come from the Mo^6+^. As shown in Figure S3c, the peak at 530.18 eV was ascribed to the lattice oxygen (O_latt_) bonds of metal oxides and the peak at 531.28 eV might be owing to the defect site of low hypoxia coordination, indicating that Ovac were created on the surface of CoMoO_4_. Figure S3d shows the C1s: the peak at 284.8 eV was ascribed to the C-C, the peak at 286.4 eV was ascribed to the C-N, and the peak at 289 eV was ascribed to the O-C=O. Figure 12c shows that the ratios of Co^3+^, Co^2+^ are 36.56% and 33.51%, and 63.44% and 66.49% for reused CMO-8 and after reuse CMO-8, respectively. Figure 12d shows that the ratios of O_latt_, O_vac_ are 72.739% and 73.817%, and 27.261% and 26.183% for reused CMO-8 and after reuse CMO-8, respectively. The Co^2+^ and O_va_ were increased by 4.277% and 7.327%, and 5.034% and 3.956% compared with the initial CMO-8, respectively. Based on this study, the repeatability of the CMO-8 catalyst is excellent.

### 3.4. MS Analysis of Oxidation Intermediates from CR

CR is easily soluble in water, with -NH_2_ and SO_3_^−^ groups in its molecular formula, just as phenol may be hydrolyzed in water, such as: SO_3_^−^ + H_2_O → HSO_3_ + OH^−^ [61].

**Figure 13:**
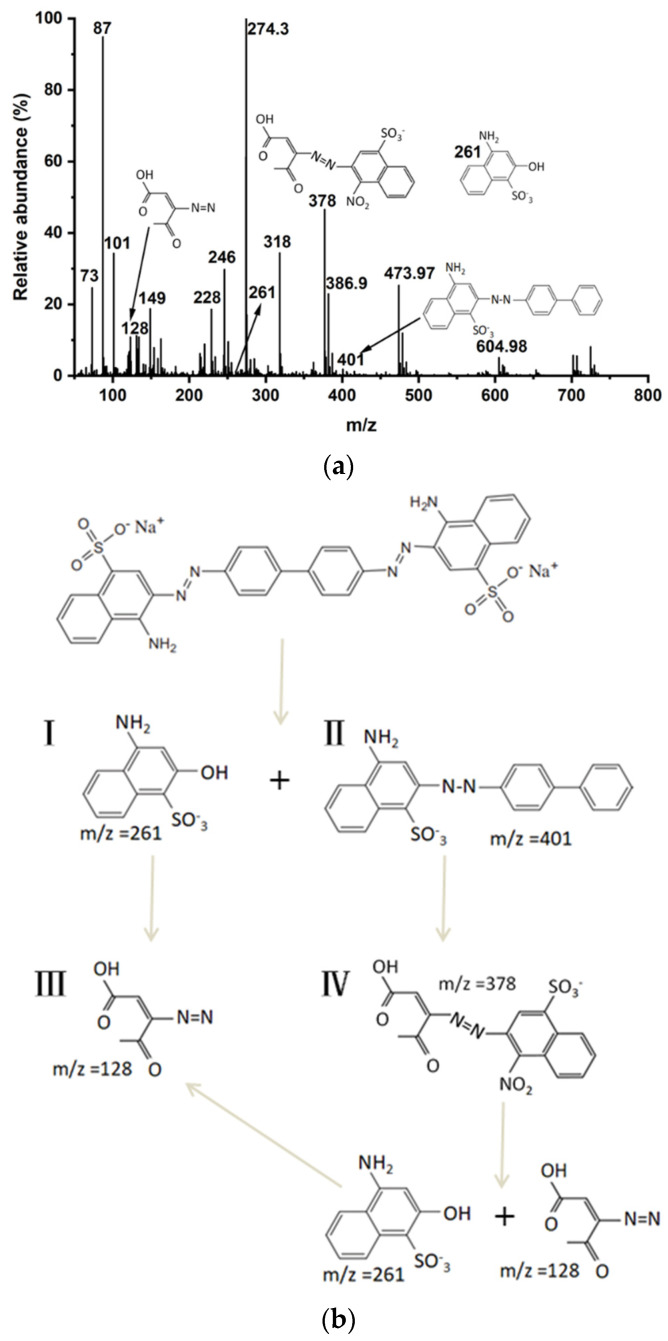
(**a**) MS spectra of Congo red dye solution after treatment with catalyst; (**b**) Product formation from the degradation of CR dye.

Figure 13 shows the degradation pathways of CR by ·OH were established from the pattern of degradation of the dye and nature of the compounds formed. Since no peak corresponding to the molecular ion peak of the dye was obtained in Figure 13a, degradation of the dye was thus confirmed. The MS method, as reported above, was used for the analysis of organic compounds of CR. CR is attacked by ·OH at the N attached to the small moiety bearing NH_2_ and SO_3_^−^, undergoes electron transfer and bond cleavage reactions, and forms products III and IV through the loss of N_2_ group [62,63,64]. Product IV under the ·OH persistent attack is composed of products I and III; these intermediates are less toxic molecules compared with Congo red dye. Additionally, other than these peaks, several more peaks of negligible intensity are present in the spectrum, indicating that most of the Congo red dye molecules have been mineralized. Although several by-products are expected to form in the dye oxidation, the identification of all the products becomes difficult due to their low solubility in methanol [63].

## 4. Conclusions

In this study, a CoMoO_4_ photocatalyst was successfully synthesized by the coprecipitation method and calcination method. The CoMoO_4_ primary product could be synthesized quickly and efficiently by the coprecipitation method. α-CoMoO_4_ with high activity was further synthesized by the calcination method, and PDS activated the CR-simulated wastewater under UV-vis. The removal rate of Congo red was 96.972% after 35 min of light exposure, and 70.27% after the 6th re-use of the material. The excellent catalytic activity of CMO-8 was attributed to the synergistic effect of photocatalysis and Co^2+^-activated PDS degradation. The capture experiments showed that superoxide radical (·O_2_), singlet oxygen (^1^O_2_), sulfate (SO_4_^−^), hole (h^+^), and hydroxyl (OH^−^) were the main free radicals leading to the degradation of CR, which was also indicated in EPR. The effects of initial concentration, catalyst dosage, PDS dosage, and pH on CR degradation efficiency were systematically investigated. The stability of both performance and structure reflected the good cyclability and stability of the reaction system. Therefore, CMO-8 catalysts can be used for the efficient removal of dye organic wastewater.

## Figures and Tables

**Table 1:** Photocatalytic performance of the prepared CoMoO_4_ in comparison with literature.

Photocatalyst	Light Source	Dye (mg/L)	Degradation (%)	Time (min)	Ref
CoMoO_4_@CMS	UV-vis	SDM 30 mg/L	98	10 min	[29]
CoMoO_4_-Co_3_O_4_	UV-vis	DBT 2000 ppm	100	180 min	[31]
CoMoO_4_	UV-vis	4-CP 50 mg/L	88	275 min	[32]
CoMoO_4_	UV-vis	MB 100 mg/L	100	40 min	[33]
CuNi/CoMoO_4_	UV-vis	AF 15 mg/L	99.45	40 min	[34]
CoMoO_4_	UV-vis	CR 100 mg	96.975	35 min	This work

## Supplementary Materials

The following supporting information can be downloaded at: https://www.mdpi.com/article/10.3390/molecules27248642/s1, Figure S1: XPS spectra of survey; Figure S2: (**a**) Effect of reused CMO-8 loading; (**b**) Effect of PMS concentration; (**c**) Effect of pH; Figure S3: XPS spectra of (**a**) Co 2p; (**b**) Mo 3d; (**c**) O 1s; (**d**) C 1s of CMO-8 reuse and CMO-8 after reuse.; Figure S4: XPS spectra of survey of CMO-8 reuse and CMO-8 after reuse.

## Sample Availability

Samples of the compounds are not available from the authors.
