# Supplementary material for: Efficient Degradation of Congo Red in Water by UV-Vis Driven CoMoO4/PDS Photo-Fenton System
Source: Molecules. 2022 Dec 7;27(24):8642. doi: 10.3390/molecules27248642 (PMC9784357; doi:10.3390/molecules27248642)
Supplement: Supplementary file 1 [file molecules-27-08642-s001.zip › molecules-2027910-supplementary.pdf]

Supporting Information

Efficient Degradation of Congo Red in Water by UV-vis Driven $CoMoO_4$/PDS Photo-Fenton System

Huimin Zhou [1], Yang Qiu [1], Chuanxi Yang [2,*], Jinqiu Zang [2], Zihan Song [2], Tingzheng Yang [2], Jinzhi Li [3], Yuqi Fan [1], Feng Dang [4] and Weiliang Wang [2,*]

1 Institute of Environment and Ecology, Shandong Normal University, Jinan 250358, China
2 School of Environmental and Municipal Engineering, Qingdao University of Technology, Qingdao 266525, China
3 Middle School of Gantian, Chenzhou 424400, China; 17674130624@139.com
4 Key Laboratory for Liquid-Solid Structural Evolution and Processing of Materials, Shandong University, Jinan 250061, China
* Correspondence: yangchuanxi1989@hotmail.com (C.Y.); sdqcsdnu@163.com (W.W.); Tel.: +86-0532-85071262

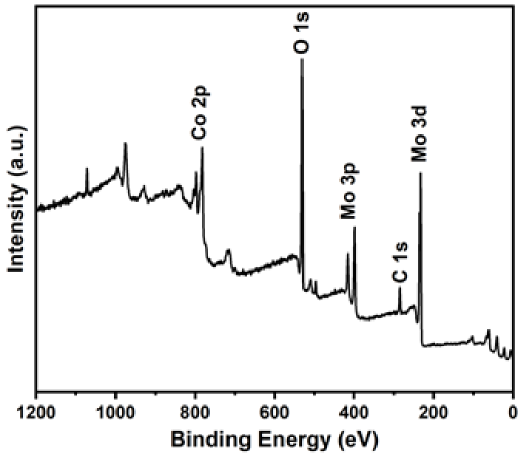

Figure S1. XPS spectra of survey.

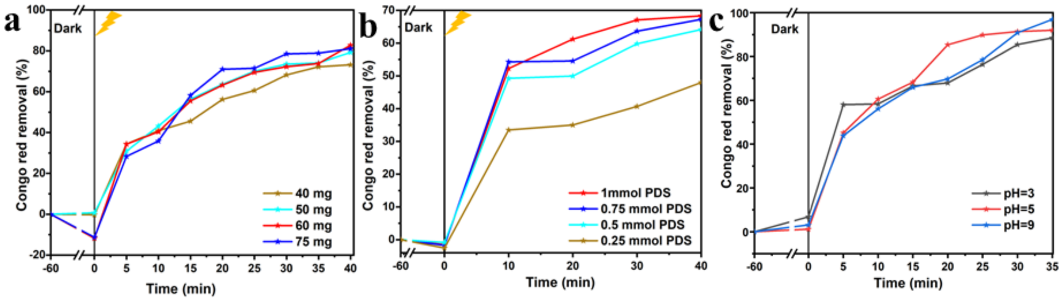

Figure S2. (a)Effect of reused CMO-8 loading; (b) Effect of PMS concentration; (c) Effect of pH

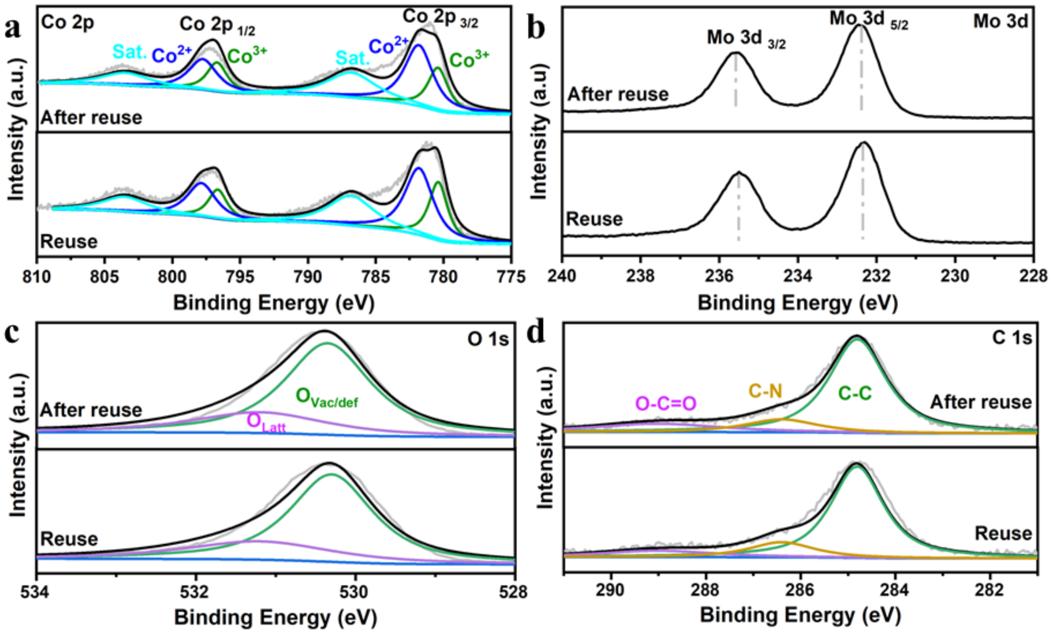

Figure S3. XPS spectra of (a) Co 2p; (b) Mo 3d; (c) O 1s; (d) C 1s of CMO-8 reuse and CMO-8 after reuse.

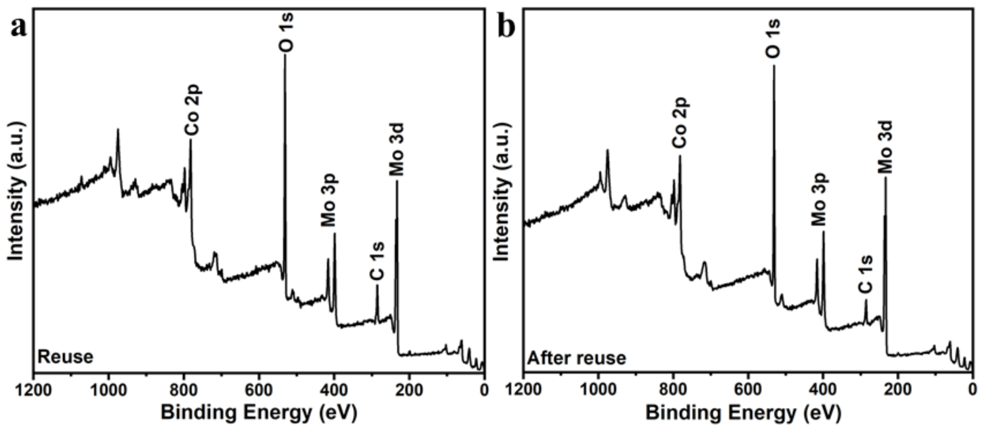

Figure S4. XPS spectra of survey of CMO-8 reuse and CMO-8 after reuse.
